# Correction: The UK Coronavirus Job Retention Scheme and diet, physical activity, and sleep during the COVID-19 pandemic: evidence from eight longitudinal population surveys

**DOI:** 10.1186/s12916-022-02502-1

**Published:** 2022-07-30

**Authors:** Bożena Wielgoszewska, Jane Maddock, Michael J. Green, Giorgio Di Gessa, Sam Parsons, Gareth J. Griffith, Jazz Croft, Anna J. Stevenson, Charlotte Booth, Richard J. Silverwood, David Bann, Praveetha Patalay, Alun D. Hughes, Nishi Chaturvedi, Laura D. Howe, Emla Fitzsimons, Srinivasa Vittal Katikireddi, George B. Ploubidis

**Affiliations:** 1 Centre for Longitudinal Studies, UCL Social Research Institute, University College London, Gower St, Bloomsbury, London, WC1E 6BT UK; 2 MRC Unit for Lifelong Health and Ageing, University College London, Gower St, Bloomsbury, London, WC1E 6BT UK; 3 MRC/CSO Social & Public Health Sciences Unit, University of Glasgow, Glasgow, UK; 4 Institute of Epidemiology and Health Care, University College London, London, UK; 5 MRC Integrative Epidemiology Unit, University of Bristol, Bristol, UK; 6 Centre for Genomic and Experimental Medicine, University of Edinburgh, Edinburgh, UK

**Correction: BMC Med 20, 147 (2022)**

**https://doi.org/10.1186/s12916-022-02343-y**

After publication, it came to the authors’ attention that 143 individuals from ALSPAC G0 and 24 in ALSPAC G1 were incorrectly coded as “unemployed” pre-pandemic in our manuscript [1].

The following are a list of corrections to the original manuscript:

i. We said: Across most studies approximately 3% of participants were no longer employed during the pandemic (8% in ALSPAC G0). Stable unemployment ranged in prevalence between 1% (GS) and 9% (ALSPAC G0).
  This should read: Across most studies approximately 3% of participants were no longer employed during the pandemic (10% in ALSPAC G0). Stable unemployment ranged in prevalence between 1% (GS) and 6% (MCS).

ii. We said: These analyses indicated that furlough was associated with increases in fruit and vegetable consumption (RR=1.22; [1.04-1.43]; I2=52%), time spent exercising (RR=1.19; [1.04-1.35]; I2=75%) and hours of sleep (RR=1.62; [1.39-1.90]; I2=80%) relative to stable employment.
  This should read: These analyses indicated that furlough was associated with increases in fruit and vegetable consumption (RR=1.22; [1.04-1.43]; I2=52%), time spent exercising (RR=1.19; [1.04-1.36]; I2=76%) and hours of sleep (RR=1.63; [1.39-1.91]; I2=80%) relative to stable employment.

iii. Figure 1 has been updated:

**Fig. 1:**
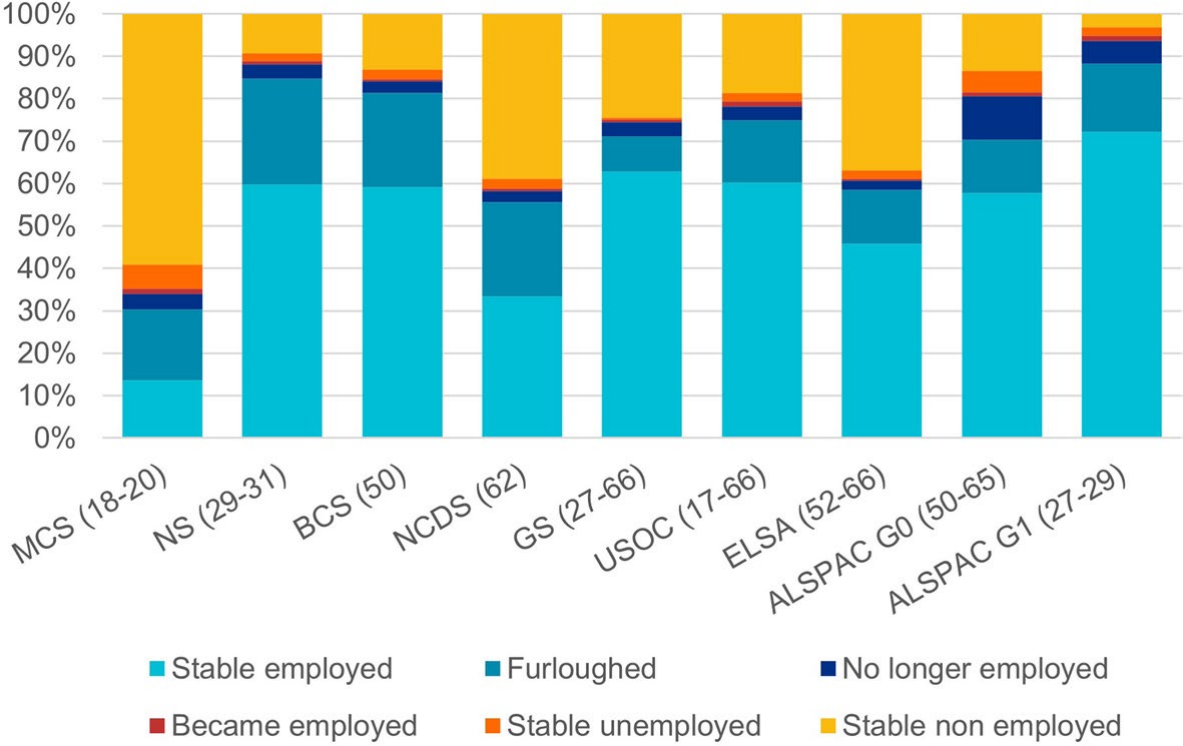
Percent distribution of change in employment status during the pandemic by study.

iv. Results in additional files 1, 3 and 4 have been updated.

The corrections in this erratum do not influence any original conclusions in this study. We apologize for any inconvenience or misunderstanding that the errors may have caused.

## Supplementary Information

**Additional file 1.**

**Additional file 3.**

**Additional file 4.**
